# A novel and effective EUS training program that enables visualization of the learning curve: Educational Program of Kindai system (EPOK)

**DOI:** 10.1016/j.vgie.2022.01.014

**Published:** 2022-03-05

**Authors:** Shunsuke Omoto, Mamoru Takenaka, Fauze Maluf-Filho, Masatoshi Kudo

**Affiliations:** 1 Department of Gastroenterology and Hepatology, Kindai University Faculty of Medicine, Osaka-sayama, Japan; 2 Department of Gastroenterology, University of Sao Paulo, Sao Paulo, Brazil

**Keywords:** EPOK, Educational Program of Kindai system

## Abstract

Video 1 A novel training method for endoscopic ultrasound operators, the Educational Program of Kindai system enables visualization of a trainee’s learning curve and difficult-to-learn areas. This visualization helps both the trainer and the trainee to structure learning and teaching methods in real time.

A novel training method for endoscopic ultrasound operators, the Educational Program of Kindai system enables visualization of a trainee’s learning curve and difficult-to-learn areas. This visualization helps both the trainer and the trainee to structure learning and teaching methods in real time.

EUS is currently regarded as a tool that enables not only observation but also diagnosis and treatment. The basis of all EUS-related procedures is the EUS screening technique. However, it can be challenging for trainees to master the technique. Several studies have investigated training methods for EUS screening that are based on memorization of typical EUS images.^1^ Hands-on training also is considered useful.^2^, ^3^, ^4^, ^5^ The American Society for Gastrointestinal Endoscopy guidelines suggest that at least 225 hands-on EUS procedures are required to achieve competency in biliopancreatic EUS.^6^, ^7^, ^8^, ^9^ However, the experience of numerous practical sessions does not guarantee the acquisition of techniques, owing to individual differences in learning.

Herein, we demonstrate a novel and effective EUS training program that enables visualization of the learning curve of a trainee in every training session through evaluation of whether a typical EUS image could be identified and how it was identified (Video 1, available online at www.giejournal.org). This program was designed to screen for biliopancreatic organs and does not include measures for mediastinal screening.

The Educational Program of Kindai (EPOK) is a unique systematic screening protocol with a scoring system. The EPOK uses 20 typical EUS images (KINDAI 20) that represent anatomical landmarks: 11 from the stomach, 7 from the duodenum bulb (D1), and 2 from the duodenum second portion (D2) (Figs. 1 and 2). These 20 stations were selected based on the assumption that the 20 EUS images cover the entire pancreatic gland and extrahepatic biliary tree. Before starting EUS training, trainees were instructed to study the EUS stations for 1 to 2 months, with reference to an atlas and EUS recordings. After commencing EUS training, the trainer provided only verbal instructions to the trainees and scored them according to the score sheet during each EUS examination (Fig. 3).

**Figure 1:**
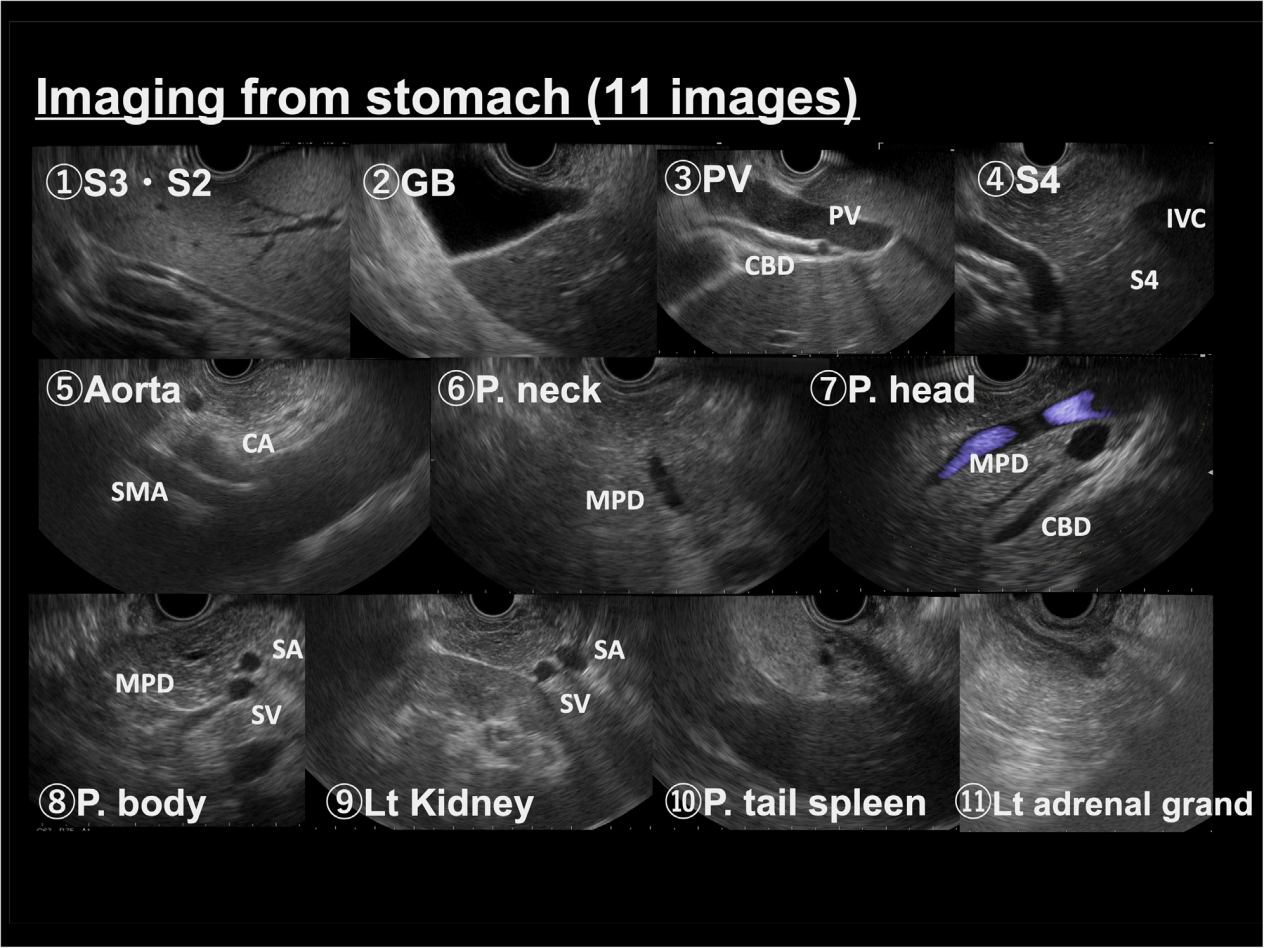
Systematic protocol for EUS screening of typical EUS images in stomach observation. The Educational Program of Kindai system includes 20 EUS images. Observations from the stomach include 11 images.

**Figure 2:**
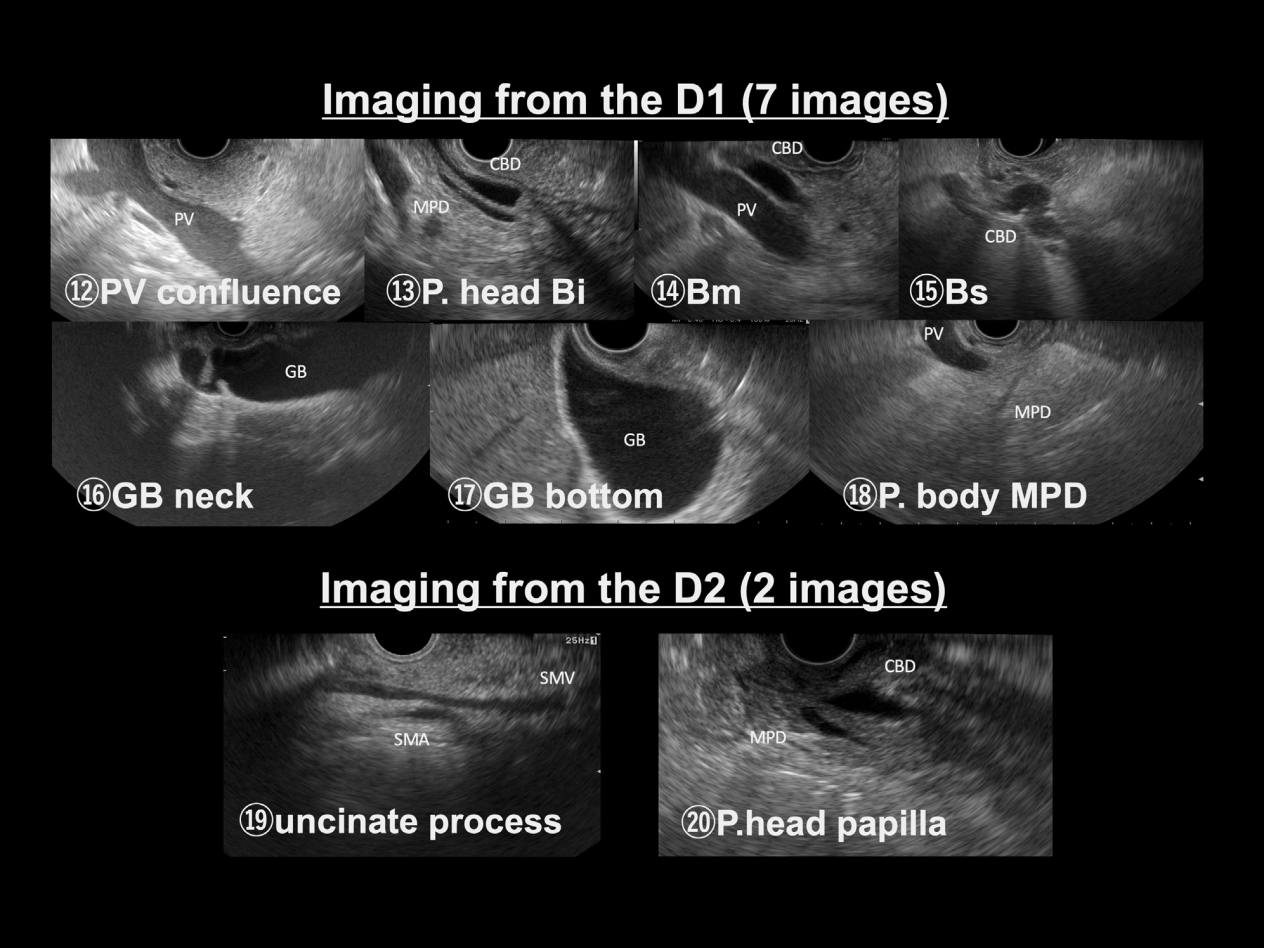
Systematic protocol for EUS screening of typical EUS images in the duodenum (D1 and D2). The Educational Program of Kindai system includes 20 EUS images. The observations from D1 and D2 include 7 and 2 images, respectively.

**Figure 3:**
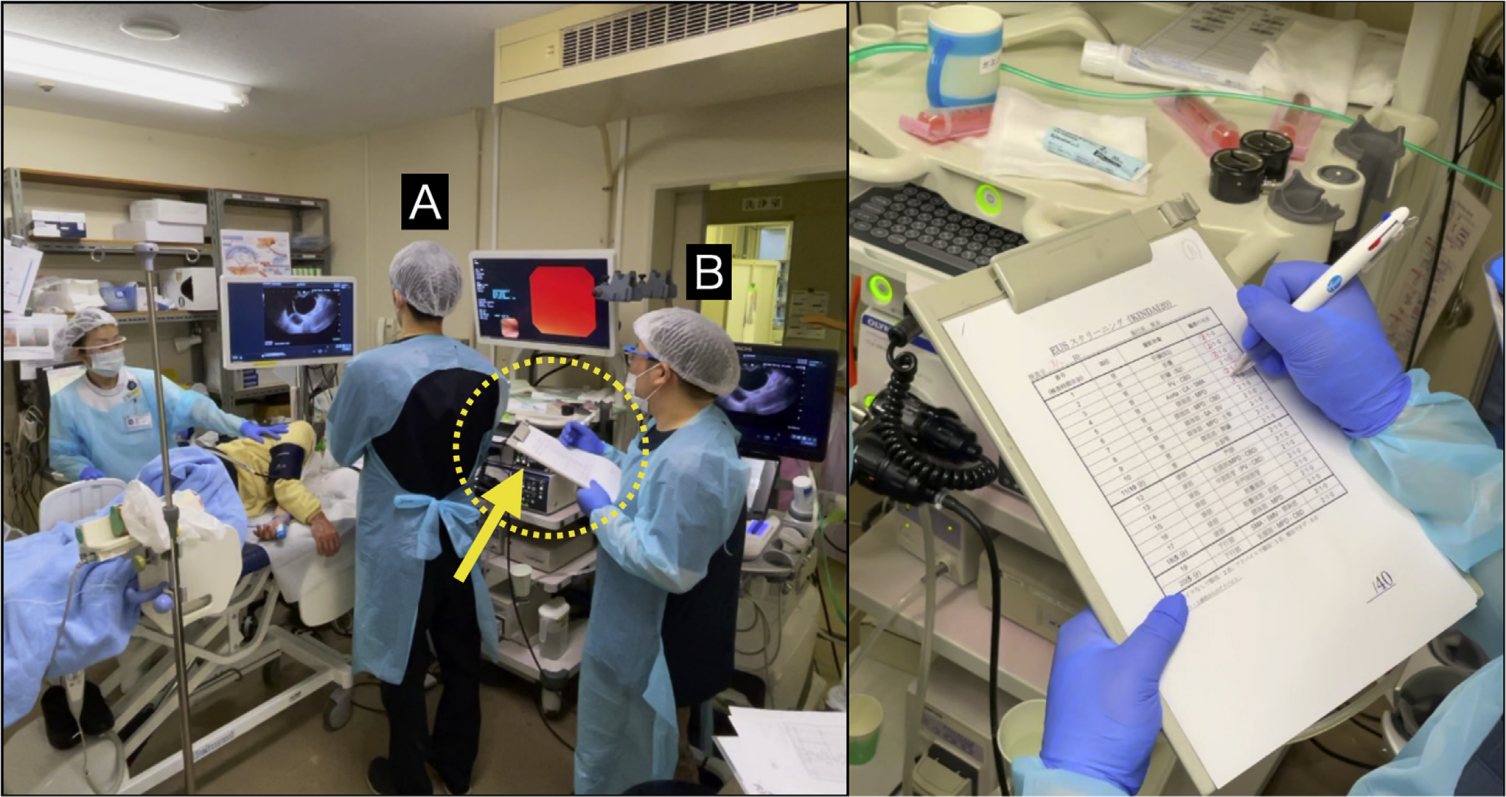
Educational Program of Kindai system of EUS. During each EUS examination, the trainer **(B)** issued only verbal instructions to the trainee **(A)** and scored the trainee according to the score sheet.

The left section of the score sheet shows the protocol number and the observation site (eg, No. 1–11 are for stomach observation), and the right section of the sheet shows the trainee’s score for each part. If the trainee could detect the image without instruction, the score was 2; if the trainee could detect the image after receiving instruction, the score was 1. If the trainee could not detect the image even with instruction, the score was 0 (Fig. 4). If the trainee detected all 20 images without instructions, 40 points were awarded. In EPOK, proficiency in EUS screening was defined as achieving a perfect score (40 points) in 3 separate examinations.

**Figure 4:**
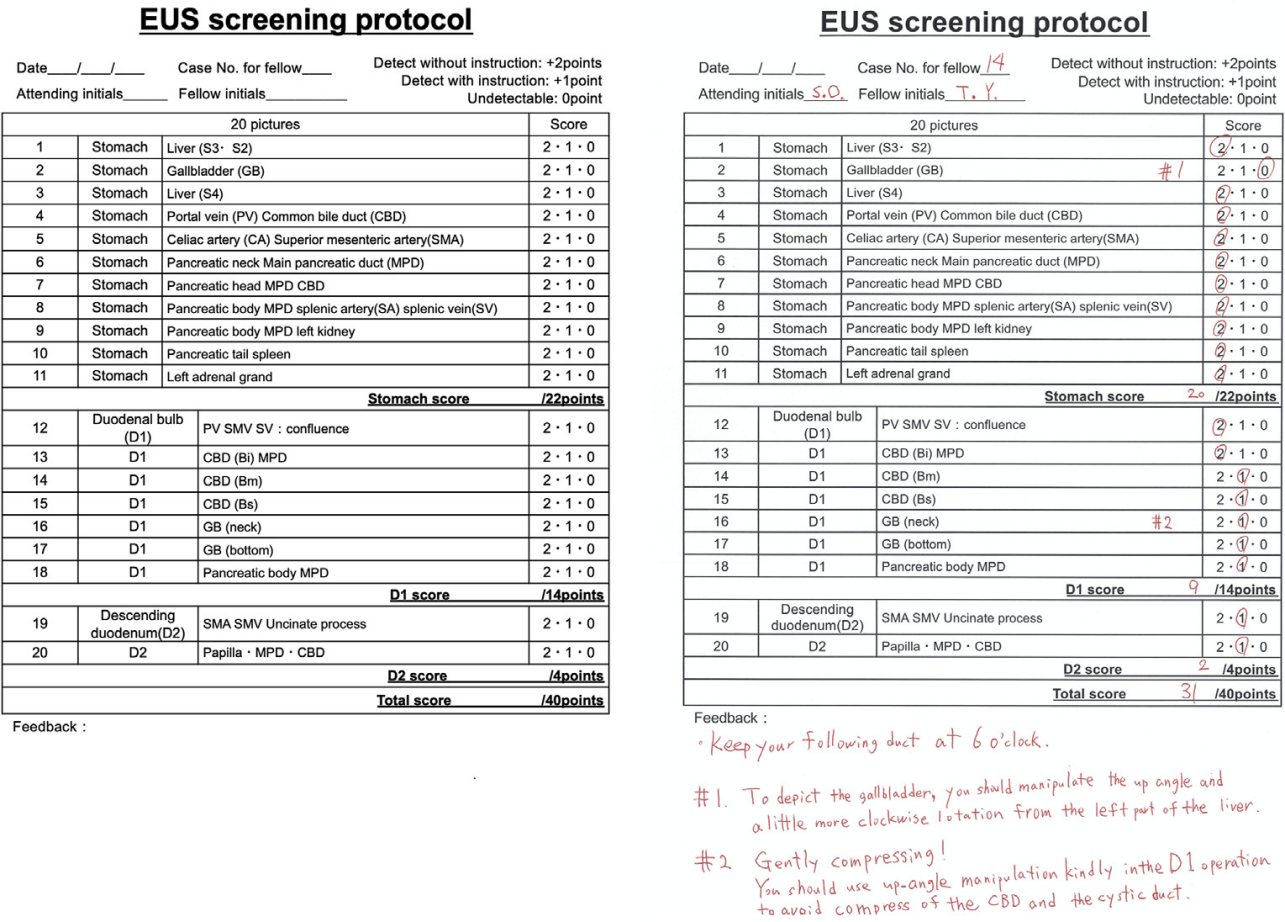
The score sheet of the Educational Program of Kindai system. The score sheets were used to calculate a total score for each examination attempt by a trainee. The Educational Program of Kindai system scores the ability of a trainee to identify each image with or without instruction. The maximum score possible is 40 points, with 2 points allocated for identification without instruction, 1 point with instruction, and 0 points for no detection. In this case, the points for each site were 20 for the stomach, 9 for D1, and 2 for D2, giving a total of 31 points. Feedback must be collated in the notes section of the score sheet after each test.

Score sheets were used to calculate the total score for each examination a trainee performed. By regularly plotting a graph of the individual’s total scores, the learning curve of the trainee can be visualized, and motivation to aim for a perfect score can be generated (Fig. 5).

**Figure 5:**
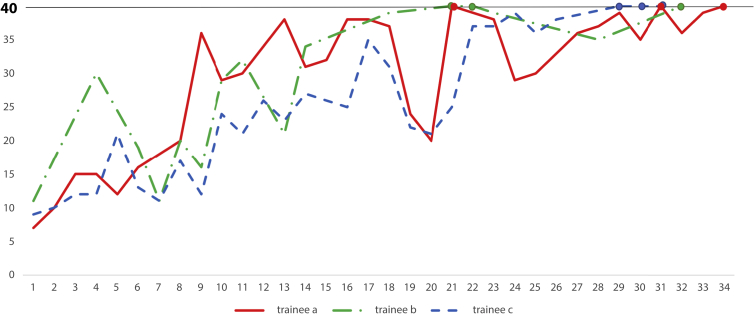
Graph showing the scores of the 3 trainees from the beginning of their Educational Program of Kindai system training to achieving their third perfect score. This graph represents each trainee's learning curve.

The trainer provides feedback after each test, recording it in the notes section of the score sheet. The feedback indicates to the trainee “what to watch out for” and “what to fix” in the next examination. The trainee is required to review the reasons for low scores (0/1) and prepare for the next examination.

In summary, although multicenter prospective studies are required to confirm the reliability and reproducibility of the scoring system, it seems notable that the EPOK enables the visualization of a trainee’s learning curve, pointing out difficult areas. This visualization helps both the trainer and trainee to structure learning and teaching methods in real time.

## Disclosure

*All authors disclosed no financial relationships.*

## Supplementary data

Video 1 A novel training method for endoscopic ultrasound operators, the Educational Program of Kindai system enables visualization of a trainee’s learning curve and difficult-to-learn areas. This visualization helps both the trainer and the trainee to structure learning and teaching methods in real time.
